# Spirituality for Coping with the Trauma of a Loved One’s Death During the COVID-19 Pandemic: An Italian Qualitative Study

**DOI:** 10.1007/s11089-021-00989-8

**Published:** 2022-02-15

**Authors:** Gianmarco Biancalani, Claudia Azzola, Raluca Sassu, Cristina Marogna, Ines Testoni

**Affiliations:** 1 Department of Philosophy, Sociology, Education and Applied Psychology (FISPPA), University of Padova, Padova, Italy; 2 Department of Journalism, Public Relations, Sociology and Psychology, Lucian Blaga” University of Sibiu, Sibiu, Romania; 3 Emili Sagol Creative Arts Therapies Research Center, Faculty of Social Welfare and Health Sciences, University of Haifa, Haifa, Israel

**Keywords:** Spirituality, COVID-19 pandemic, Death, Grieving process, Funeral rite

## Abstract

Spirituality may be a key factor in reducing the negative psychological effects of traumatic events and a means by which the experience of grief can be processed.

The objective of the present research is to assess whether and how spirituality provided concrete support in those who lost a loved one during the COVID-19 pandemic. The participants are 8 people from the most affected cities in northern Italy. They were interviewed in depth, the interviews were transcribed and the texts were analyzed through Interpretive Phenomenological Analysis. The results show that spirituality has been found to be a protective factor with regard to the processing of grief in crisis situations such as the COVID-19 pandemic, in particular with regard to the belief that the deceased loved one is now in an otherworldly dimension. In addition, the celebration of a funeral rite offers support to the grieving person in the early stages of mourning thus laying the foundation for a healthy grieving process. It is therefore important to support individual spirituality, which can be a useful tool for processing the traumatic experience, especially in difficult times such as the COVID-19 pandemic.

The experience of loss after the death of a significant person causes bereavement, which is a psychological state of all-encompassing grief. The depth of suffering is important and often causes significant negative consequences in the daily life of the grieving person (Attig, 1996; Testoni, 2015. This suffering requires the activation of weighty grief work to gain a new existential normal beyond negativity, anxiety, and distress (Cohen & Johnson, 2017; Gonçalves et al., 2015; Hai et al., 2019; Newman & Graham, 2018; Wass, 2004). One of the factors that allows the grief to be better processed is the spiritual dimension (Diener et al., 2011; González-Sanguino et al., 2020; Suryani et al., 2011). Many studies confirm that those who grieve through the spiritual dimension suffer less from loneliness and are more resilient (Angell et al., 1998; Cadell et al., 2012; Damianakis & Marziali, 2012; Walsh, 2007, 2020). Despite the pain that grief entails, particularly traumatic grief, it is possible to transform loss into an opportunity for growth (“post-traumatic growth”; Bonanno et al., 2004; Tedeschi & Calhoun, 2004, 2008) since some people manage to transform trauma and difficulties into opportunities for change and personal development (Páez et al., 2012; Seybold, 2007; Walsh, 2020). One of the factors enabling this transformation is spirituality, which facilitates post-traumatic growth processes (Koening, 2020; Prieto-Ursua & Jodar, 2020).

The current situation caused by COVID-19 has forced most of the world’s population to change their lifestyles, and one of the areas to have undergone major change is death, including both individual and community bereavement (Aguiar et al., 2020; Testoni et al., 2021). Indeed, some factors of the current COVID-19 pandemic may make grief work more difficult: sudden death, patient isolation, the severity of COVID-19 symptoms, the rapidity of the inauspicious course of the disease, lack of social support (Crispim et al., 2020), social isolation (Banerjee & Rai, 2020; Menichetti Delor et al., 2021; Saltzman et al., 2020; Stroebe & Schut, 2020; Walsh, 2020), and widespread feelings of helplessness (Kokou-Kpolou et al., 2020). One of the most significant factors inhibiting the spiritual dimension is the impossibility of accompanying the deceased in a regular course of funeral rituals (Carr et al., 2020; Otani et al., 2017). In particular, COVID-19 deaths have been sudden and unexpected deaths that have not been followed by the usual rituals that help the bereaved to activate good grief work (Aguiar et al., 2020; Ruini et al., 2003; Walsh, 2020). The literature has already shown how the absence of this aspect makes grief work more complex because the mourner feels a lack of social support and symbolic sharing (Franqueira, 2019; Park & Halifax, 2011).

The aim of the present research was to assess whether and how spirituality provided concrete support to those who lost a loved one during the COVID-19 pandemic.

## Methods

### Participants

The participants were eight individuals aged between 23 and 54 (see Table 1) from different northern Italian cities most severely affected by COVID-19 (Senni, 2020). They were recruited in 2020 using convenience sampling. Participants were contacted by researchers through an Italian Facebook group for people who had lost loved ones because of COVID-19. The number of participants is appropriate for qualitative research, according to the standards required for a qualitative methodology (Denzin & Lincoln, 2005; Guest et al., 2006).

**Table 1:** Participants

Pseudonym	Age	Who they lost	Time between death and interview	Religious belief
Elia	50	Mother and father	3 months	Practicing Christian
Cecilia	23	Grandmother	5 months	Practicing Christian
Angelica	30	Father	2 months	Practicing Christian
Fabio	29	Father	2 months	Atheist
Nicola	48	Father	5 months	Atheist
Lorenzo	54	Mother	4 months	Nonpracticing Christian
Daniela	44	Father	3 months	Nonpracticing Christian
Serena	32	Father	4 months	Practicing Christian

### Data Collection and Analysis

Each participant was interviewed by Skype or Zoom, with an average of 60 min per call. The interview method was explained in advance to the participants, and they were asked to choose which platform was most convenient for them. The interviews were conducted in a synchronous online mode that, in accordance with the literature (Allen, 2017; Hanna, 2012), allowed the researchers to collect information from a geographically wider population than would be possible otherwise. Even though the literature shows that this interviewing mode may limit the possibility of establishing a relationship of trust, as happens during in-person interviews, because the use of a computer that stands between the interviewer and the interviewee can be seen as a virtual barrier (Mann & Stewart, 2000), this limitation was not detected during the interviews for the present study. In fact, conducting the interviews in a personal place such as their own home facilitated an openness among the participants even towards sensitive issues.

The aim of the interview was to explore the resources participants drew on to cope with their grief and the role of the spirituality when dealing with the trauma of not being able to physically be with or say goodbye to their loved one. The interview focused on how each participant experienced the pandemic and the associated difficulties. They were also asked what strategies they implemented to cope with the traumatic event and how spirituality was crucial to them in this process.

The interview method was explained in advance to the participants. The conversations were recorded and transcribed in preparation for analysis. All the texts were analyzed using a qualitative thematic analysis that focuses on recognizing key meanings and concepts (Braun & Clarke, 2012). The thematic analysis was conducted according to the six phases outlined by Braun and Clarke (2012): familiarization with the data, coding, generating initial themes, reviewing themes, defining and naming themes, and writing up the report. Specifically, a reflexive thematic analysis was used that allowed allows researchers to change, remove, and add codes as they work through the data not using a codebook (Braun & Clarke, 2013). The reflexive thematic analysis is theoretically flexible and well suited for studying participants’ experiences and perceptions (Braun & Clarke, 2012). The approach that was adopted is inductive; it involves the process of coding and creating themes from the data without any preconceptions (Boyatzis, 1998).

The texts were processed using Atlas.ti software (Muhr, 1991), which allows researchers to work directly on written texts, highlight portions of them, create labels to be inserted in each text to adequately represent the fundamental themes, and construct larger clusters of meaning by comparing the data obtained from each text (Testoni, Iacona, et al., 2018). Four of the researchers who conducted this study offered their expertise in qualitative data analysis and code identification.

## Ethics Statement

The research followed APA Ethical Principles of Psychologists and Code of Conduct and the principles of the Declaration of Helsinki, so all the objectives of the research and the methodology of analysis used were explained in detail to the participants. Given the emotional difficulty of the topic, we reiterated to the participants that they could stop the interview at any time they wished and without giving any explanation if they needed to. We asked them for permission to record the conversations, to transcribe their answers, and to analyze their content. We also guaranteed that we would anonymize the content of the transcripts. Only those who had given written and signed consent participated in the research. This study was approved by the Ethics Committee for Experimentation of the University of Padova (approval 27331296116C7206D8A5B61A06F4845B).

## Results

From the analysis of the results, two main themes emerged that were consistent with the objectives of the research: “the importance of funeral rites for grieving” and “spirituality as a support in the elaboration of mourning.”

Theme 1: The importance of funeral rites for grieving.

Restrictions during the first wave of the pandemic made it impossible for family members to accompany their loved ones during their illness and suffering, and normal funeral services were not available. Many families last saw their loved ones when they were being put into an ambulance. Later, they were given a funeral urn containing the ashes of their loved one. In fact, for most of the participants it was not possible to organize a funeral rite, nor was it possible to share hugs of consolation and comfort with relatives and friends. The participants’ narratives made explicit the pain resulting from the lack of a funeral rite, which was necessary to help them start processing their grief. Daniela, a 44-year-old woman who lost her father, said,

I can’t fully realize that my dad has died. Although I have received the ashes, it doesn’t seem real to me that my father is dead. The ashes are not my father. It’s like there’s a piece missing, a passage. Where is my father’s body?

Not being able to perform a funeral rite was one of the factors hindering the grieving process, locking the mourners into a kind of denial of the event. Cecilia, a 23-year-old woman, when telling us about her grandmother’s death, said,

We prayed at the cemetery, but it was just us, there were four of us and none of the relatives, aunts, and uncles could join us for the lock-in. Even the four of us couldn’t hug and embrace. We met each other at a distance of five meters without being able to touch each other. It is difficult to say what we experienced and to find the words to describe the pain of this experience. It was all so unreal, incomprehensible. I cannot accept what happened and everything still seems unreal to me.

A few months after her father’s death, Serena, a 32-year-old woman, and her family were asked by the village priest to hold a memorial service. She said, “We were all there and we had a memorial service, but obviously it’s not like a proper funeral, when you sanctify the body and you imagine that the blessing of your loved one allows him to continue beyond death.”

The mourning phase, as can be seen from the testimonies above, is a key step in the grieving process as it allows a full realization of the painful event, leading to greater awareness and consequently a greater ability to manage the emotions and experiences that characterize the loss of a loved one.

Angelica, a 30-year-old woman, interprets this absence from a regulatory point of view:

There was neither a mortuary nor a guarantee that it was my father who was in the coffin. We didn’t know who was inside the coffin they showed us, and we managed every step with two other coffins outside the cemetery. For the burial, it was just us: my mother and us children. We prayed quickly and buried him where the gravestone is now. But that rite was not a funeral. You can’t call that moment a funeral. It was something else.

The same incredulity was experienced by Cecilia:

Not having seen with my own eyes and not having lived close to her when she needed me, I cannot think that she is dead. I still imagine her there, and it seems unreal that I don’t visit her. But knowing that she is dead makes it even more difficult.

Elia, a 50-year-old man who lost both parents, experienced the lack of the rite of passage as violence: “This death for me was an affront to my father. To leave like that without saying goodbye to anyone, without being accompanied by children and friends, without a ritual in church, is not right. My father did not deserve this death.”

The lack of a funeral ritual prevented the mourners from paying their last respects to their loved ones, and this resulted in a lack of full realization of the event, generating a situation of strong disbelief, even regarding the recognition of the deceased, because the participants did not have the opportunity to see their loved one either before or after their death.

In contrast, the experience of being able to perform the funeral rite allowed the mourners to face the experience of separation and loss with a sense of reality. Lorenzo, a 54-year-old man, recounted, “We all felt the need to have this rite to pay our respects, to accompany her, to be among relatives.” He described the pain caused by the impossibility of accompanying his mother in the last days of her life, but being able to celebrate the funeral, he said, “was liberating because it was a way to accompany her and to let her know that we were there and that we had never abandoned her.” The funeral was a means of recovering the relationship they had missed out on with their mother in her moment of extreme need: “The celebration of the funeral, even though it was not normal, took on a special flavor because we wanted to meet and be close to my mother in some way, recovering the experience we were unable to have when she died.”

Lorenzo’s words show that it is of fundamental importance to be able to organize a funeral (even long after the event) as a farewell, a commemoration, and a reunion and to be able to recover, even after some time, that closeness, that human warmth that was made impossible by the rules in force during the pandemic.

Theme 2: Spirituality as a support in the elaboration of mourning.

Religions have always had a central role with respect to death because they offer perspectives that enable humans to make sense of dying, thus opening a window to the meaning of a painful experience. Elia recounts being received at the Vatican after writing a letter to the pope, using a religious metaphor:

After the meeting with Pope Francis, I understood that either you lose your faith or you strengthen it. COVID-19 puts you to the test. In any case, you ask yourself, ‘Why me?’ Then I think of Jesus Christ, who went into the desert for 30 days and 30 nights alone. That’s how I felt: either you lose Christ or you assimilate Him.

In Elia’s opinion, as for other participants, the negative experience of COVID-19 strengthened their faith in Christ because Christ represents someone who goes through the most atrocious sufferings and the deepest humiliations, including death, and comes out victorious. Christ, from their point of view, teaches humans to fear neither pain nor death. Reliance on prayer and Christ’s teachings made it possible to process negative emotions more deeply and to feel safe despite the abnormal and tragic context. Christ, therefore, becomes the figure who teaches how to transform anger into action in order to seek a solution and change the situation for the better. The transformation of the negativity of anger into strength to deal with pain thus takes on the appearance of the deepest mysticism, rather than a cognitively oriented process. Elia, speaking of his own emotions of anger, said,

It is right to get angry! But I experienced my anger as a special form of prayer. Because someone who doesn’t get angry, who doesn’t cry and doesn’t feel anything, is someone who doesn’t give a damn about what is happening. They don’t care about the pain of others. And that is not good! Getting angry is a form of prayer, and one should not be ashamed of one’s indignation and the need to cry out in anguish. You have to vent the pain you have inside because suffering belongs to all human beings and you have to be able to recognize it. If you are angry, you want to change the situation.

For Elia, the imitation of Christ allowed him to live out his anger entirely as if this emotion were a prayer. The potentially destructive feeling thus took on a positive value and was not repressed but consciously managed, thanks to having faith in Christ. Almost all of the participants described something similar, although less deeply and emphatically than in Elia’s narrative.

The anger of the participants was related to the poor management of information with respect to the risk of infection by COVID-19, the mismanagement of care by the health service, the inability to accompany their loved ones towards the end of life, and the actual death of their relatives. For some participants, these causes of grief and anger resulted in a loss of faith. One example is Nicholas, a 48-year-old man who, unlike Elia, fell into a deep existential crisis:

Right now, I am so angry about my father’s death that I cannot think that he is with God. I imagine him as dust because of the cremation they had to do to his corpse. I am a Catholic Christian by choice, but now that choice is no longer supported by faith. Someone has to explain to me what kind of God would allow this. I do not understand at all what divine plan this pandemic corresponds to. How can God allow all this tragedy? Now, I represent God as a Nazi. If I have to believe in God and believe that He exists, I can only portray Him as negative because I don’t understand how He can allow all this.

Another typical aspect of Catholic Christianity that has helped mourners to better cope with grief is the representation of the afterlife. In particular, the representation of heaven was characterized by most of the participants as mitigating their suffering caused by the pain of not being able to support their loved ones during their final illness, as Serena said:

I think my father is in heaven and now he is my guardian angel. I don’t know where he is, but I know that he is in a better place and that he can see us from there. I am convinced that our thoughts can reach him and that he now knows that we did not abandon him when he was sick and when he was dying. He knows that I’ve always loved him and that I suffered when I couldn’t reach him in hospital and how much I suffered when they told me he was dead.

Angelica also found great strength and comfort in the belief that her father now lives in an afterlife dimension:

I don’t believe in God so much as I believe in the afterlife. In fact, I feel my father close to me, and when I suffer I feel him even more present. I’m aware that it’s a matter of faith and that not everyone shares these beliefs or doesn’t believe that my father can stay close to me even after death. Yet it is precisely this thought that helps me to go on, to find the strength to face the difficulties that this pandemic imposes on us. I think of my father and want to imagine him in a better place. I think he is better off than he was here on earth, and I imagine him happy with those he loved who died before him.

Faith and the spiritual dimension, when they were not undermined, helped to keep the emotional bond alive and reassuring. They also helped those grieving to make sense of the loss and the pain they were suffering, as Elia explained:

I am sure that all these victims of COVID, who suffered unspeakably, are now close to God because they have become angels. In heaven, they needed good people and they called them. I cannot think otherwise. To think that this might not be true makes me sick.

Fabio, a 29-year-old man, said, “This is a time of pain, but we must not escape this experience; instead, we must understand what the victims of COVID-19 have taught us.”

## Discussion

The present study is in line with previous research regarding the importance of the role played by spirituality in grieving (Cadell et al., 2012; Diener et al., 2011; Gonçalves et al., 2015; González-Sanguino et al., 2020; Hai et al., 2019; Newman & Graham, 2018; Suryani et al., 2011; Wass, 2004). The research was carried out in an area severely affected by the first wave of the pandemic in northern Italy. Among the participants, the importance of spirituality emerged, which was manifested as the maintenance of an empathic relationship with the deceased or continuing bonds made possible by imagining the deceased’s existence in the afterlife. Being able to think about the deceased in the afterlife allowed participants to find comfort and help in coping with what they were experiencing and, in agreement with Cohen and Johnson’s (2017) assertion, gave them a way to feel less alone and to think that they were understood in their grief. Catholic religiosity differs from spirituality in that, as already highlighted in the literature (Pargament, 2001), it allows believers to recognize Christ as the fundamental example to refer to in order to make sense of their pain. In particular, as clearly manifested by Elijah’s narrative, Christ is the reference figure needed to transform the negative emotions caused by stress and mourning for those who died during the pandemic into the positive actions required to change the situation and not become indifferent. Most participants therefore found spirituality and religiosity to be a resource for strengthening their resilience. A virtuous circle was then established in them, whereby their experience of resilience strengthened their faith.

However, this was not the case for everyone. On the contrary, the shock caused by the sudden deaths and the pain of the losses caused a deep crisis of faith in some, for whom God came to be depicted as a “Nazi.” These issues have been emphasized by Exline and Rose (2005) in connection with religious and spiritual struggles. Religion and spirituality provide powerful sources of comfort, direction, and meaning for many people, but they can also be sources of tension and struggle. Many individuals experiencing great loss, as during the COVID-19 pandemic, feel angry with God. Although people try to maintain their faith and virtue in accordance with their beliefs, sometimes—as in this period—this has not been enough (Exline, 2013).

The literature has already considered the problem and refers to the exquisitely psychological phenomenon resulting from representing one’s God as a figure of attachment and explaining the tragedies of life as due to abandonment by God (Cohen & Johnson, 2017; Exline, 2013). The representation of God as an abandonment figure inevitably causes further suffering, together with the inability to transform anger into a resilient force (Bjorck & Thurman, 2007; Lee et al., 2013). This phenomenon is accompanied by the loss of the ability to represent death as a transition to an afterlife. Consistent with research conducted by Testoni et al. (2016), Testoni et al. (2017), Testoni, Bisceglie, et al. (2018)), people who view death as annihilating the deceased report greater states of anxiety in dealing with issues related to their own and others’ deaths than those who view death as a transition, which also affects the resources for resilience that fuel their ability to cope with the pain of loss and the possibility of being able to find meaning in their loss (Salsman et al., 2015; Testoni et al., 2015, 2017). With respect to the ability to find meaning in their loss, and in line with Tedeschi and Calhoun’s (2004, 2008) research and Walsh’s (2020) recent studies on resilience during the pandemic, some participants stated that they sought comfort in the belief that their loved one’s death had a greater purpose and should be a teaching. This need to entrust a noble and humanitarian meaning to the death of their loved one gave them relief. Studies show that this strategy for dealing with traumatic grief reduces stress and the risk of the grieving person developing complicated grief (Bekkering & Woodgate, 2019; Bellet et al., 2018; Cardoso et al., 2020; Neimeyer & Sands, 2011).

In addition, we found that those who were able to perform a funeral ritual, even long after the loved one’s death, greatly benefited from it, recovering the sense of social closeness that was denied by the harsh restrictions due to COVID-19. Funeral rites and prayer, vigil, or burial allow for accompaniment and support to be offered to the grieving person in the early stages of mourning, thus laying the foundation for a healthy grieving process (Park & Halifax, 2011). Finally, those who were not able to accompany their loved one and give them a regular funeral service were greatly affected. This experience also caused strong feelings of disbelief (Hamid & Jahangir, 2020; Mortazavi et al., 2020; Testoni et al., 2020) and difficulty adjusting to reality (Carr et al., 2020; Kokou-Kpolou et al., 2020; Walsh, 2020). This phenomenon was closely related to the inability to see the body. In fact, some studies have described the importance of having direct contact with the corpse of the deceased in order to complete the mourning process (Testoni, Iacona, et al., 2018).

## Conclusion

The grief experienced during the first wave of the COVID-19 pandemic was a real challenge for the research participants and put them to the test emotionally as they had feelings of grief and anger at the same time.

The inability to say a final goodbye, to see the body of the deceased with their own eyes, and to celebrate the body severely affected their ability to peacefully deal with the loss. However, spirituality and religiosity proved to be protective factors in the face of such a profound crisis, providing support for most of the mourners. On the other hand, those who had to cope while feeling psychologically abandoned by the God in whom they believed had more difficulty with anger management. Our results, therefore, confirm the importance of these dimensions and how important it is that they mature over the course of people’s lives in such a way that they can support people in times of difficulty.

This study has many limitations due to the fact that there were few participants. One of the limitations is about the recruitment of the participants; only those who were using social networks during the pandemic could take part in the research. In the future, it would be advantageous to recruit participants in different ways and to expand the number of participants, to better understand the phenomenon.

In addition, it would be useful to investigate through a follow-up whether faith continued to fuel the resilience of the believers over time, including whether those who reported losing their faith due to the pandemic had problems grieving.

## Data Availability

Not applicable.
